# Opioid legislation and narcotic filling in total hip arthroplasty: descriptive study of time and state-level trends in the United States

**DOI:** 10.1186/s13011-021-00410-w

**Published:** 2021-09-28

**Authors:** Daniel J. Cunningham, Sean P. Ryan, Steven Z. George, Brian D. Lewis

**Affiliations:** 1 Department of Orthopaedic Surgery, Duke University Medical Center, 200 Trent Drive, Durham, NC 27710 USA; 2 Duke Clinical Research Institute, 200 Morris Street, Durham, NC 27701 USA

**Keywords:** Opioid, State legislation, Total hip arthroplasty

## Abstract

**Background:**

The opioid misuse epidemic focused national attention on reducing opioid overprescribing. The purpose of this study is to describe the relationship of time and state-level interventions and opioid filling surrounding total hip arthroplasty (THA) in the United States.

**Methods:**

A national database with diverse insurance constituents was queried for first-prescription and cumulative perioperative opioid filling volumes and rates in oxycodone 5-mg equivalents (OE’s) in 487,942 patients undergoing primary THA from 30-days pre-operative to 90-days post-operative. Descriptive statistics evaluated pre-legislative and post-legislative opioid filling by state, legislative type, and surgery year.

**Results:**

At the national level, initial opioid filling volumes have remained largely unchanged (56.2 OE’s in 2010 to 51.7 OE’s in 2018). Meanwhile, cumulative opioid filling volumes (151.9 OE’s in 2010 to 111.7 OE’s in 2018) have decreased considerably. Rates of initial opioid prescriptions exceeding 90 OE’s were similar in 2010 (6.4%) and 2018 (5.6%). States with legislation targeting duration and volume of opioid prescriptions saw the largest decreases in opioid prescription filling. That is, 75% of states with opioid legislation had large (> 10 oxycodone 5-mg equivalents) decreases in cumulative 90-day opioid filling compared to only 20% of states without opioid legislation having large decreases in cumulative 90-day opioid filling.

**Conclusions:**

This descriptive study demonstrates decreases in perioperative opioid filling for THA. Although this study was descriptive in nature, states enacting opioid-limiting legislation had larger decreases. Although causal relationships could not be inferred from this analysis, the results suggest that states without legislation could improve prescriber compliance with national goals of decreased opioid overprescribing by enacting opioid-limiting legislation.

**Level of evidence:**

Level III, retrospective prognostic cohort study.

**Supplementary Information:**

The online version contains supplementary material available at 10.1186/s13011-021-00410-w.

## Introduction

Total hip arthroplasty (THA) is an invasive procedure, and despite excellent long-term outcomes and improvement in function, initial post-operative pain can be difficult to manage [1]. Throughout the 1990’s there was a significant effort to focus on controlling patients’ post-operative pain. In 1999, the California legislature mandated recording of patient’s pain along with vital sign measurements [2]. This newfound focus on pain as a vital sign in the 2000’s, in conjunction with research demonstrating that pain is one of the most important determinants of satisfaction following THA [3], led to widespread increased utilization of opioids in the perioperative period.

Opioids continue to be commonly used to control immediate post-operative pain. However, more recently, the opioid epidemic has garnered significant attention as the number of opioid related deaths and chronic opioid users continue to grow [4]. Amongst interventions leading to chronic opioid use, spine and orthopaedic procedures have stood out as the top two drivers for initial prescriptions that lead to sustained opioid use [5]. Thus, some legislators have made efforts to amend opioid prescribing practices and instituted maximum prescribing limits. For arthroplasty surgeons in particular, this has been an area of active research and discussion throughout the American Association of Hip and Knee Surgeons (AAHKS), in an effort to investigate and change opioid prescribing habits.

The purpose of this study is to describe the relationship of time and state-level interventions and opioid filling surrounding total hip arthroplasty (THA) in the United States.

## Method

### Study design

This was a descriptive study of perioperative opioid filling in patients undergoing primary total hip arthroplasty between 2010 and 2018 using a large, national database. This study was designed and reported in accordance with the Strengthening the Reporting of Observational Studies in Epidemiology (STROBE) statement on observational studies [6]. This study was approved by the institutional review board.

### Variables and data sources

All patients ages 18 and older undergoing primary total hip arthroplasty were identified in the PearlDiver (PearlDiver, Inc.) Mariner dataset (see Additional file 1: Table S1 for codes used to identify primary total hip arthroplasty) and opioid filling trends evaluated from 30-days pre-operative to 90-days post-operative. This dataset includes information on 122 million distinct patients from 2010 to 2018 with a broad geographic and demographic variety of insurance coverage (Medicare, Medicaid, commercial, and cash-pay) in the United States. The database facilitates blinded longitudinal, patient-specific tracking of information available through International Classification of Disease (ICD)-9, ICD-10, and Current Procedural Terminology (CPT) codes. Importantly, this database keeps record of patients across states, providers, and care settings. Further, it includes information on all prescriptions filled at all pharmacies within the United States with exception of inpatient pharmacies. All opioids were included in this study with exception of opioids intended for cough suppression, which were identified by presence of alpha-agonists or anti-histamines. In line with recent recommendations on opioid-related database studies in orthopaedic surgery, this specific dataset was selected due to the granular, patient-level information that it provides on opioid filling [7]. Although this dataset provides users exceptional granularity, researchers are blinded to the data and are limited in statistical testing to preserve anonymity. For that reason, a descriptive approach was utilized. In order to select a cohort of patients with broad applicability to patients undergoing primary total hip arthroplasty for osteoarthritis, exclusion criteria included prior hip fracture diagnosis (see Additional file 1: Table S1 for codes used to exclude hip fracture) and patients with exceptionally high perioperative opioid demand (> 8000 morphine milliequivalents or 467 oxycodone 5-mg equivalents or OE’s filled from 1-month pre-operative to 3-months post-operative). The cohort included all patients with active insurance status from 6-months pre-op to 3-months post-op, *n* = 541,353. The main study outcomes were the volume of opioids filled and rate of opioid filling and refills over the study timeframe. Filled prescriptions were converted from oral morphine equivalents (OME’s) into oxycodone 5-mg equivalents (OE’s) for ease of interpretation using conversion factors proposed by the Centers for Disease Control (CDC) [8]. Baseline patient and operative factors were recorded including age, sex, obesity (see Additional file 1: Table S1 for coding definition), Charlson comorbidity index (CCI), pre-operative (6-months to 1-month pre-operative) opioid filling, year of surgery, and state of opioid prescription. Patients that filled opioid prescriptions within the 6-month to 1-month pre-operative window were dichotomized into patients with one versus two or more prescriptions. The patients with two or more prescriptions were considered to have chronic opioid use, similar to definitions used by the CDC. However, the 1-month pre-operative period was excluded since some patients may have received and filled an opioid prescription pre-operatively intended for post-operative usage [9].

Next, opioid prescribing legislation was reviewed for each state (see Additional file 2: Table S2, [10]). Some states limited opioid duration, volume, or both factors. Additionally, some states passed legislation that was specific to certain small subgroups of patients such as patients on Medicaid. In this analysis, only legislation that affected all state residents was considered. Legislation dates were determined for each state and opioid filling was evaluated before and after legislation. Some states have not enacted legislation. In this case, September 10, 2017 was used as the before/after date since it was the mean date of legislation passage in the cohort of states in which opioid legislation was passed. Lastly, year-specific rates of initial prescriptions exceeding 90 OE’s (675 MME’s) were also evaluated to determine the relationship of time and outlier prescribing. 90 OE’s was chosen to represent a “large” opioid prescription based on a prior study [11].

### Data analysis

A descriptive approach was selected rather than an inferential approach. Trends were clear and statistical comparisons were not particularly useful in setting of the exceptionally large sample size. Further, advanced statistical testing such as multilevel modeling, which could address anticipated year-level and state-level clustering, is not available within the blinded dataset and statistical environment that PearlDiver offers. Baseline characteristics and outcomes were displayed with means (standard deviations), medians, or proportions (percentages) as appropriate. Unadjusted outcomes were calculated including mean OE’s per filler and rates of one or more or two or more opioid prescriptions. Prescription filling data were broken down by year, legislation presence/absence, legislation type, and state to further analyze these factors.

### Funding

There were no sources of funding for this study.

## Results

Most patients were 65 and older (Table 1). Males comprised 43% of the population. 20% of patients were obese. 23.4% of patients filled two or more opioid prescriptions within the 6-month pre-operative to 1-month pre-operative timeframe. The median CCI was 0, while the mean was 1.1. The distribution of patients across the 2010–2018 timeframe was even with consideration of fewer patients that were able to meet active insurance status at the end of the dataset’s available timeframe (2018) and fewer patients available for analysis in 2010.

**Table 1:** Baseline patient and operative characteristics

Baseline patient and operative characteristics	Study cohort (***n*** = 541,353)
*Age*	
18 to 19 years	185 (0%)
20 to 24 years	713 (0.1%)
25 to 29 years	1274 (0.2%)
30 to 34 years	2149 (0.4%)
35 to 39 years	4214 (0.8%)
40 to 44 years	8927 (1.6%)
45 to 49 years	19,830 (3.7%)
50 to 54 years	41,043 (7.6%)
55 to 59 years	66,534 (12.3%)
60 to 64 years	85,568 (15.8%)
65 to 69 years	91,048 (16.8%)
70 to 74 years	122,581 (22.6%)
75 to 79 years	93,717 (17.3%)
80 to 84 years	3570 (0.7%)
Male sex	234,875 (43.4%)
Obesity	109,291 (20.2%)
*Pre-operative opioid use*	
One pre-op opioid prescription	79,174 (14.6%)
Two or more pre-op opioid prescriptions	126,792 (23.4%)
*Charlson comorbidity index*	
CCI 0	289,815 (53.5%)
CCI 1	109,118 (20.2%)
CCI 2	62,630 (11.6%)
CCI 3	34,447 (6.4%)
CCI 4	17,597 (3.3%)
CCI 5 or more	27,746 (5.1%)
Mean CCI (SD)	1.1 (1.77)
*Year*	
2010	21,718 (4%)
2011	61,833 (11.4%)
2012	66,077 (12.2%)
2013	70,591 (13%)
2014	75,123 (13.9%)
2015	74,112 (13.7%)
2016	73,574 (13.6%)
2017	69,798 (12.9%)
2018	28,514 (5.3%)

As shown in Table 2, initial opioid filling volume has not changed dramatically (56.2 OE’s in 2010 to 51.7 OE’s in 2018). However, the overall cumulative (151.9 OE’s in 2010 to 111.7 OE’s in 2018) opioid filing volume has decreased 26% since 2010. This drop appeared to be most dramatic between 2016 and 2018. Rates of initial opioid prescription volume exceeding 90 oxycodone 5-mg equivalents increased from 2010 (6.4%) to 2015 (10.3%) and then decreased to 2018 (5.6%).

**Table 2:** Year-specific opioid filling volumes and rates across the entire study cohort in oxycodone 5-mg equivalents. Mean (standard deviation) or sample size and percentage displayed

Timeframe	Oxycodone 5-mg equivalents (first prescription)	Oxycodone 5-mg equivalents (cumulative)	1 or more opioid prescriptions	2 or more opioid prescriptions	Initial volume > 90 oxycodone 5-mg equivalents
2010	56.2 (38.9)	151.9 (105.3)	12,917 (59.5%)	8171 (37.6%)	1387 (6.4%)
2011	55.8 (38.7)	141.8 (105.7)	39,590 (64%)	26,208 (42.4%)	4319 (7%)
2012	56.8 (38.6)	140.8 (104.9)	42,277 (64%)	27,684 (41.9%)	4901 (7.4%)
2013	57.5 (38.3)	140.2 (104.5)	46,734 (66.2%)	30,102 (42.6%)	5666 (8%)
2014	58.7 (38.8)	139.8 (102.4)	50,784 (67.6%)	32,575 (43.4%)	6508 (8.7%)
2015	62.2 (39.9)	140.4 (101)	50,271 (67.8%)	31,286 (42.2%)	7622 (10.3%)
2016	61.5 (39.6)	136.8 (99.7)	51,373 (69.8%)	31,429 (42.7%)	7311 (9.9%)
2017	57.7 (37.3)	126.5 (96.1)	49,009 (70.2%)	29,448 (42.2%)	5619 (8.1%)
2018	51.7 (33.9)	111.7 (91.3)	19,943 (69.9%)	11,484 (40.3%)	1590 (5.6%)

When compiling states with and without legislation and evaluating initial and cumulative opioid prescriptions pre-act and post-act, states with and without legislation have had reductions in opioid filling. However, states with legislation had larger magnitude of reductions in both initial and cumulative filling volume (Table 3) compared to states without legislation (9.8 vs 1.3 initial and 33.8 vs 16.8 cumulative OE’s).

**Table 3:** Pre-act and post-act initial and cumulative opioid filling in oxycodone 5-mg equivalents in states with and without legislation. Mean (standard deviation, sample size) displayed

Pooled states	Pre-act	Post-act
*Initial prescription*		
With legislation	59.6 (39.3; *n* = 251,007)	50.1 (31.9; *n* = 29,261)
Without legislation	58.1 (38.7; *n* = 60,614)	56.8 (37.3; *n* = 5024)
*Cumulative prescriptions*		
With legislation	140.2 (103.1; *n* = 373,988)	106.4 (87.9; *n* = 42,113)
Without legislation	142.5 (103.6; *n* = 93,674)	125.7 (96; *n* = 7297)

**Fig. 1:**
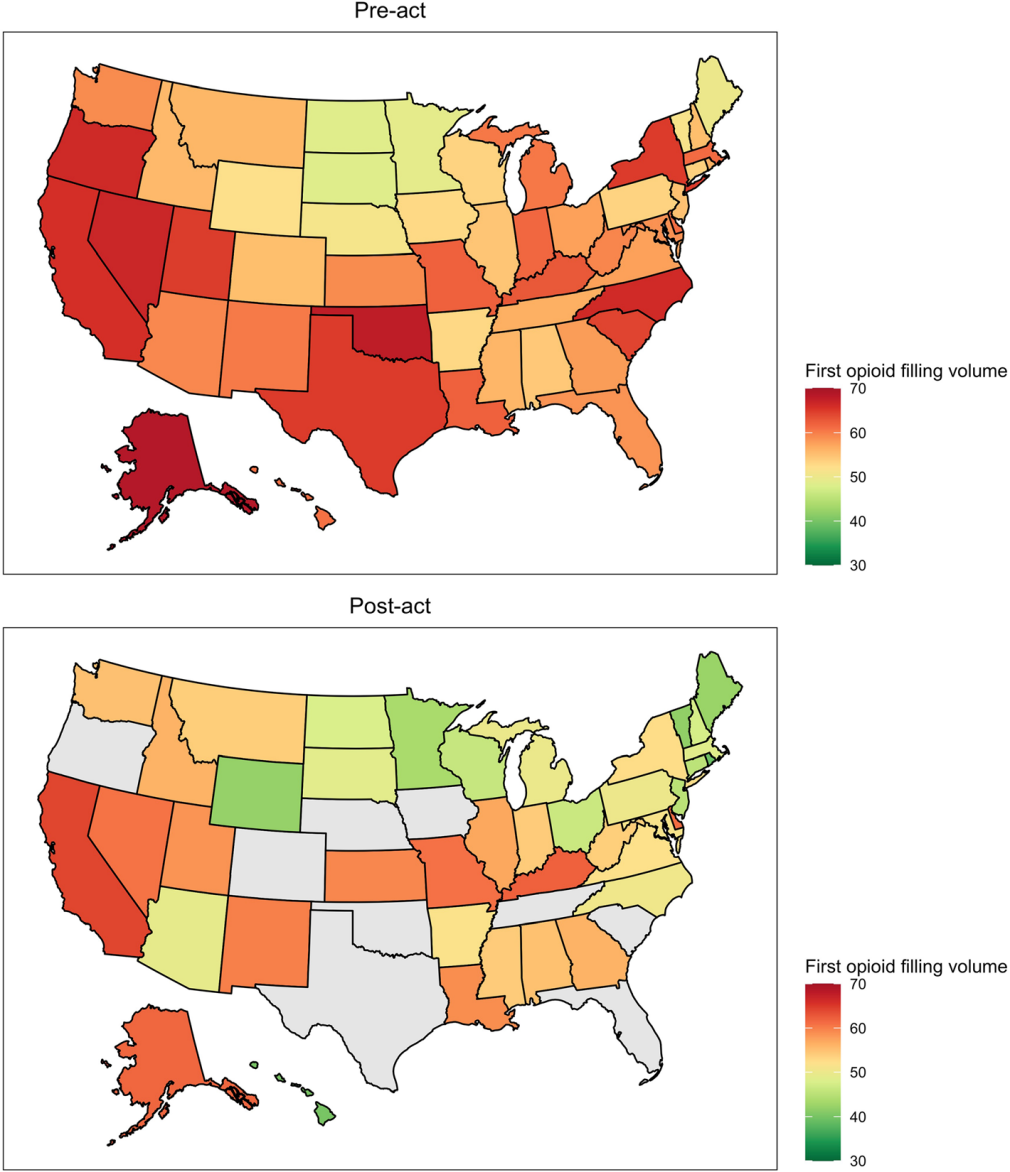
First opioid prescription volume pre-act and post-act heat map. States in grey had insufficient data post-act to display. For states without legislation, 9/10/2017 was used as the act date

**Fig. 2:**
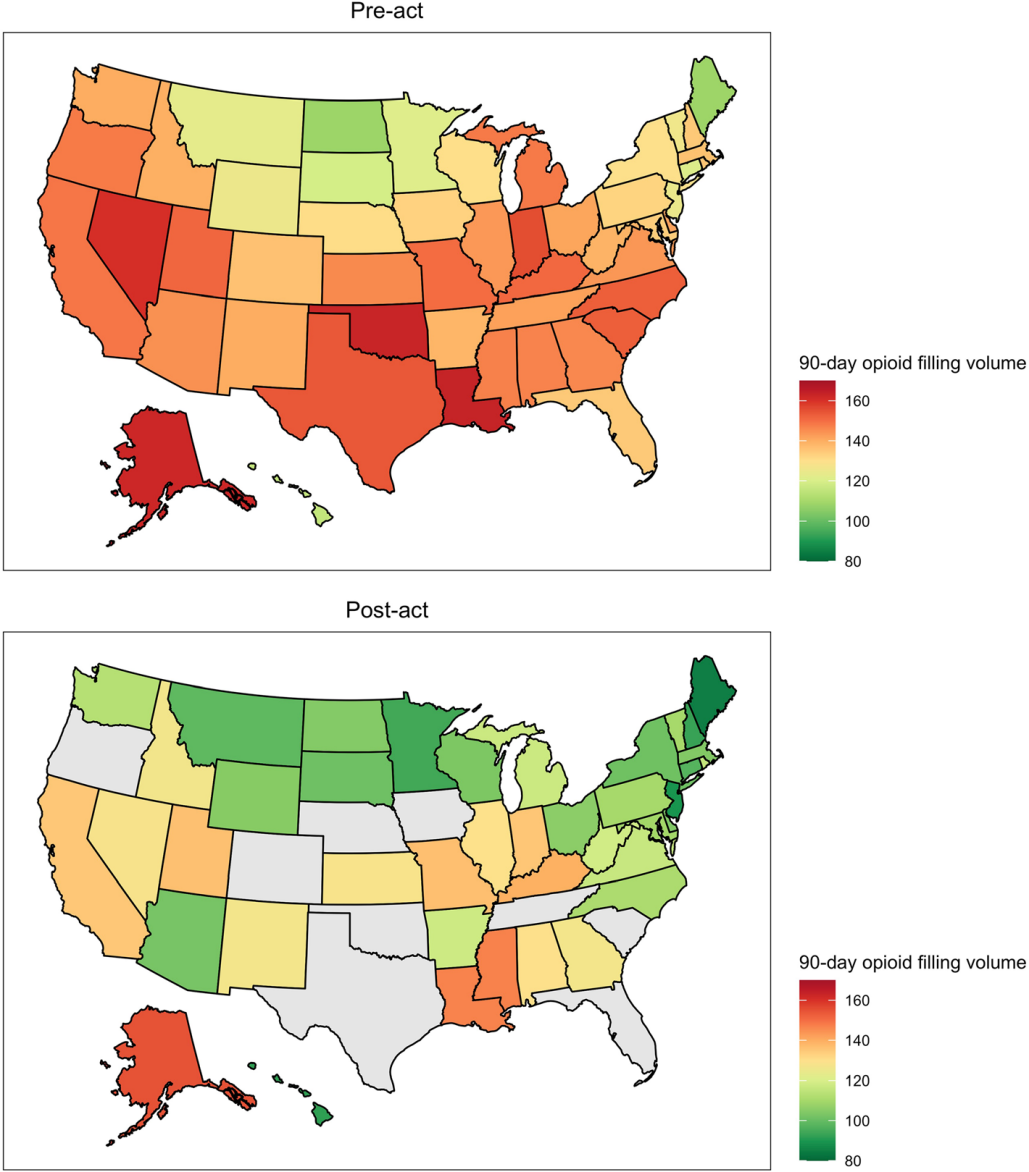
Cumulative opioid prescription volume pre-act and post-act heat map. States in grey had insufficient data post-act to display. For states without legislation, 9/10/2017 was used as the act date

States with legislation targeting duration and volume had the largest reductions in pre-act to post-act opioid filling volume (Table 4). Figures 1 and 2 show a heat map of initial and cumulative opioid filling volume before and after legislation for specific states. Appendix Figures 3 and 4 show year-level changes in state-specific opioid legislation.

**Table 4:** Initial and 90-day post-operative cumulative opioid filling volume in pre-act and post-act legislation cohorts by legislation type in oxycodone 5-mg equivalents. Means and standard deviations displayed

Legislation type	Pre-act	Post-act
*Initial*		
No legislation	58.1 (38.7; n = 60,614)	56.8 (37.3; n = 5024)
Duration	59.6 (39.7; *n* = 187,681)	50.7 (32.1; *n* = 22,757)
Volume	58.9 (37.7; *n* = 6763)	54.8 (34.3; *n* = 699)
Duration and volume	58.9 (38; *n* = 35,602)	46.1 (29.8; *n* = 4638)
No specified duration or volume	60.1 (39.1; *n* = 20,961)	51.7 (31.8; *n* = 1167)
*Cumulative*		
No legislation	142.5 (103.6; n = 93,674)	125.7 (96; n = 7297)
Duration	139.9 (103.3; *n* = 278,282)	106.7 (87.3; *n* = 32,772)
Volume	139.6 (102.2; n = 10,779)	113 (92; *n* = 1040)
Duration and volume	142.6 (103.4; *n* = 51,505)	103 (89.8; *n* = 6531)
No specified duration or volume	138.8 (100.7; *n* = 33,422)	109.9 (89.7; *n* = 1770)

Table 5 demonstrates reductions in pre-act to post-act initial and cumulative opioid filling volumes. With regard to initial prescription filling volume, differences were largest in Arizona, Hawaii, Massachusetts, Michigan, North Carolina, New York, Ohio, and Rhode Island (exceeding 10 oxycodone 5-mg equivalents). Meanwhile, while there were large reductions in cumulative opioid filling volume for many states. Notably, Arizona and New Hampshire each had reductions greater than 40 OE’s.

**Table 5:** 1-month pre-operative to 90-days post-operative initial and cumulative prescription opioid filling volume by state in oxycodone 5-mg equivalents. States with no opioid legislation were included for comparison. The mean date of legislation (9/10/2017) was used for states without opioid legislation. Mean (standard deviation, sample size) displayed. Sample sizes for initial opioid filling volume only include patients with at least one filled opioid prescription during the timeframe

State	Pre-act initial	Post-act initial	Initial difference	Pre-act cumulative	Post-act cumulative	Cumulative difference
*Duration*						
KY	62.9 (41.1; *n* = 4726)	62.3 (39.5; *n* = 751)	0.7	151 (108.5; *n* = 6942)	139.8 (104.8; *n* = 1094)	11.2
MN	49 (31.6; *n* = 5400)	43.9 (28; *n* = 901)	5.1	121.3 (96.7; n = 10,543)	94.5 (80.9; *n* = 1607)	26.7
AZ	59.4 (37.7; *n* = 7225)	49.3 (31.6; n = 251)	10.1	144.6 (106.5; n = 10,019)	102.4 (83.8; *n* = 355)	42.1
NJ	55.1 (36; n = 11,955)	45.4 (27.5; *n* = 2470)	9.7	124.5 (95.7; n = 18,023)	89.9 (75.1; *n* = 3541)	34.6
AK	68.8 (42; *n* = 564)	61.7 (42.9; *n* = 55)	7.1	162.1 (108.1; *n* = 804)	154.6 (113.3; *n* = 79)	7.4
CT	53.8 (35.1; *n* = 5103)	45.6 (26.5; *n* = 1817)	8.2	121.1 (93.7; *n* = 8447)	97.5 (81.1; *n* = 2756)	23.6
DE	61.2 (38.3; *n* = 2143)	61.5 (32.3; *n* = 379)	−0.3	141.6 (100.6; *n* = 2821)	104 (80.4; *n* = 499)	37.6
HI	60.8 (39.9; *n* = 565)	40.2 (35.4; *n* = 72)	20.6	115.8 (89.2; *n* = 990)	91.8 (96.9; *n* = 111)	24
IN	61.8 (41.3; *n* = 8802)	54.4 (33.8; *n* = 918)	7.4	155.4 (109; n = 11,731)	135.2 (98.8; *n* = 1229)	20.2
LA	62.4 (41.2; *n* = 2912)	59.1 (37.1; *n* = 366)	3.3	163.7 (111.7; *n* = 3924)	146.8 (108.9; *n* = 468)	16.9
MA	61.5 (37.5; *n* = 4644)	48.9 (31.1; *n* = 1846)	12.6	136.1 (98.9; *n* = 6875)	104.5 (86.8; *n* = 2586)	31.6
MI	60.6 (38.5; n = 16,155)	50 (31; *n* = 1035)	10.5	148.2 (107.3; n = 23,048)	117.4 (91.6; *n* = 1385)	30.8
NC	66.4 (43; *n* = 7591)	50.5 (29.9; *n* = 277)	15.9	152.7 (104.8; n = 11,605)	111.1 (87.7; *n* = 418)	41.6
NH	55.3 (33.8; *n* = 1072)	47.6 (27; *n* = 274)	7.8	134.7 (96.7; *n* = 1450)	93.1 (75.7; *n* = 369)	41.6
NY	65 (43.5; *n* = 16,052)	52.8 (34.3; *n* = 5847)	12.2	129.9 (98; n = 25,301)	101.5 (83.6; *n* = 8430)	28.4
PA	53.5 (36.2; n = 14,895)	50.3 (31.8; *n* = 4099)	3.2	132.4 (100.5; n = 22,882)	110.2 (87.8; *n* = 5739)	22.2
UT	65 (42; *n* = 1415)	59.4 (32.7; n = 145)	5.6	151.2 (106.6; *n* = 2263)	136.4 (97.5; *n* = 213)	14.8
VA	57.7 (38.3; *n* = 5826)	52 (32.2; *n* = 1224)	5.7	143.8 (103; *n* = 8585)	116.2 (91.4; *n* = 1832)	27.5
WV	59.6 (38.1; *n* = 2636)	53.8 (33.1; *n* = 28)	5.8	138.9 (104.2; *n* = 3794)	118.5 (89.9; *n* = 55)	20.5
*Volume*						
VT	51.5 (27.3; *n* = 237)	41.8 (22; n = 21)	9.7	125.4 (86.9; *n* = 405)	110 (113.2; *n* = 38)	15.4
WA	59.2 (38; *n* = 6526)	55.2 (34.5; *n* = 678)	4	140.2 (102.7; n = 10,374)	113 (91.3; *n* = 1002)	27.1
*Duration and volume*						
OH	57.7 (37; n = 24,998)	46.6 (29.3; *n* = 2646)	11.1	141.4 (102.5; n = 36,003)	105.8 (88.9; *n* = 3763)	35.6
RI	56.7 (34.1; *n* = 970)	39.6 (29.6; *n* = 421)	17.1	135.6 (98.5; *n* = 1407)	116.2 (105.3; *n* = 574)	19.5
NV	66.9 (46.2; n = 2756)	60.7 (37.7; *n* = 367)	6.2	160.5 (110; *n* = 3752)	128 (96; *n* = 483)	32.5
ME	50.1 (31.4; *n* = 1874)	42.6 (26.7; *n* = 1204)	7.4	108.6 (92; *n* = 3135)	84.7 (80; *n* = 1710)	23.9
*No specific duration or amount*						
MD	58.8 (38.1; *n* = 7721)	51.7 (31.8; n = 1167)	7.1	133.9 (99.5; n = 11,978)	109.9 (89.7; *n* = 1768)	24
*No legislation*						
AL	54.4 (38; *n* = 2898)	55.4 (33.5; *n* = 315)	−1	146.5 (110.6; *n* = 4109)	130.3 (100.1; *n* = 415)	16.2
AR	53.1 (37.3; *n* = 1688)	51.8 (36.6; *n* = 173)	1.3	138.3 (107.9; *n* = 2546)	117.6 (99.9; *n* = 231)	20.7
CA	65.9 (43; n = 12,892)	64.5 (39.9; *n* = 1013)	1.4	148.6 (106.7; n = 21,706)	135.4 (101.2; *n* = 1661)	13.1
DC	49.7 (28; *n* = 473)	52.4 (56; n = 49)	−2.8	120.3 (92.6; n = 733)	108.9 (101.1; *n* = 74)	11.4
GA	58.1 (36.7; n = 10,808)	56.4 (38.2; n = 699)	1.7	145.9 (103.1; n = 14,847)	126.8 (94.9; *n* = 974)	19.1
ID	55.9 (36.3; *n* = 774)	56.2 (41.5; *n* = 83)	− 0.4	140 (101.4; *n* = 1299)	126.6 (96.7; *n* = 119)	13.4
IL	54.7 (37.4; n = 11,995)	57.1 (39.8; *n* = 969)	−2.4	143.3 (103.6; n = 17,967)	129.6 (96; *n* = 1377)	13.7
KS	59.1 (37.7; *n* = 2143)	59.4 (36.8; *n* = 184)	−0.3	145.8 (103.2; *n* = 3183)	128.3 (100.9; *n* = 255)	17.5
MO	62.2 (42.4; *n* = 4561)	61.1 (35.5; *n* = 405)	1.1	150.1 (104.8; *n* = 6727)	136.7 (96.1; *n* = 554)	13.4
MS	56.3 (38.1; *n* = 1373)	54.1 (39; *n* = 105)	2.2	147.2 (105.5; *n* = 2062)	147 (118.2; *n* = 164)	0.2
MT	56 (33.8; *n* = 823)	54 (30; *n* = 74)	2	123.1 (96.4; *n* = 1378)	98.8 (61.5; *n* = 120)	24.3
ND	48.6 (29.4; *n* = 853)	47.8 (31; *n* = 98)	0.8	108.6 (85.8; *n* = 1396)	104.5 (78.5; *n* = 124)	4.1
NM	60.6 (38.4; *n* = 1014)	60 (41; *n* = 110)	0.6	139.8 (102.8; *n* = 1518)	126.8 (95.6; *n* = 141)	13
SD	48.4 (30.8; *n* = 1070)	49.1 (32.8; *n* = 168)	− 0.7	118.8 (88.3; *n* = 1797)	101.2 (76.8; *n* = 230)	17.6
WI	53.4 (34.5; *n* = 6770)	46.1 (24.5; *n* = 531)	7.3	130.2 (97.1; n = 11,673)	103.5 (83.5; *n* = 793)	26.8
WY	52.3 (41.8; *n* = 479)	43 (23.3; *n* = 48)	9.3	125.1 (98.4; *n* = 733)	107.1 (82.9; *n* = 65)	17.9

## Discussion

This descriptive study reports perioperative opioid filling volume and rates of opioid filling and refills after primary, THA in a large, commercially available insurance database from 2010 to 2018. The purpose of the study was to describe the relationship of time and opioid-related legislation on perioperative opioid filling. In doing so we reported general trends of opioid prescription, including identifying potential variation by state level legislations. Specifically, cumulative (152 OE’s in 2010 to 112 OE’s in 2018) opioid filling volume has decreased since 2010 while initial opioid filling volume (56 OE’s in 2010 to 52 OE’s in 2018) and the rate of initial opioid filling volume exceeding 90 OE’s (6.4% in 2010 and 5.6% in 2018) has remained largely unchanged. States with legislation targeting opioid prescription duration and volume had the largest decreases in initial and cumulative opioid prescription filling. This was a descriptive study so while these findings are compelling, they should primarily be used to generate hypotheses for future study of this important topic.

Due to the growing attention around the opioid epidemic and evidence suggesting that orthopaedic procedures may contribute to chronic opioid use, research on perioperative opioids around THA has become increasingly common [2]. Delaney et al. demonstrated that for opioid naïve patients 65 years of age or greater undergoing THA, high initial opioid prescriptions were correlated with prolonged opioid use [12], and Hannon et al. showed that when more tablets are prescribed, it was independently associated with greater consumption, despite no differences in pain scores or patient reported outcomes measures [11]. This is important, because several authors have demonstrated that institutional protocols for prescribing can directly lead to a reduction in opioid consumption [13–15]. Still, however, the optimum number of narcotic tablets to prescribe at discharge is multifactorial and likely influenced by individual institution’s multimodal analgesia protocols, patient factors, and surgeon and hospital experience. Runner et al., in a prospective cohort study, demonstrated that the average number of days taking narcotic analgesia was 8.5, with an average of 20.8 pills taken during that time (despite 72.5 pills prescribed) [16]. In a recent survey of AAHKS members, the average number of narcotics tablets prescribed was 44 (range 0–200) at discharge from THA, with 74% of respondents utilizing multimodal analgesia [17]. Indeed, there remains significant variability in opioid prescribing after THA, with higher tablet volumes coming from providers with fewer years in practice and who had higher volume practices [18].

In part, based on the growing body of evidence demonstrating perioperative opioid use as contributing to prolonged narcotics use, individual State and the Federal government have passed opioid legislation [19] enacting prescribing limits, clinical guidelines, prescription drug monitoring programs, mandated continuing education, and recovery centers. Rhode Island law to reduce opioid prescribing was passed in 2016, which limited opioid naïve patients to 30 morphine milligram equivalents per day or 20 doses post-operatively [20]. Studies in other subspecialties such as hand surgery and sports medicine have demonstrated surprising disconnects between the volume of opioid prescribed and consumed [21–23]. Reid et al. demonstrated that for opioid naïve patients undergoing arthroplasty, cumulative postoperative prescriptions were significantly decreased following enactment [20].

Our results demonstrate state-by-state variability with opioid prescribing. This study adds depth and breadth to the findings of Sabatino et al., which suggested large intra-institutional variability in opioid filling across a variety of elective orthopaedic procedures [24]. Given the growing body of legislation, the considerable down-trend in filling seen in 2016–2018 (cumulative perioperative opioid filling) across the nation may be the inflection point the opioid overprescribing epidemic has been waiting for. It is also important to recognize that states without opioid-limiting legislation have had decreases in prescription filling volumes. While impossible to prove with this dataset, the authors speculate that these reductions are related to increased social awareness of the dangers of opioids.

There are several notable limitations to this study. First, we utilized a large national database and are unable to review individual patient charts. However, in order to evaluate national opioid trends, we feel this is appropriate. Furthermore, the PearDiver database includes private insurance claims as well as Medicare, thus allowing analysis of a large patient cohort to identify subtle data trends. Second, we are unable to know the indications for opioid prescribing. It is possible that patients received opioid prescriptions for indications unrelated to their THA. We anticipate that this effect is small in the perioperative timeframe, however. Third, we do not have data on opioid consumption or opioid prescribing and are only able to report on opioid filling. While opioid consumption data would be ideal, opioid filling still may be a more accurate metric of opioid demand than opioid prescribing, as prescribing likely reflects prescriber practices to a greater degree than opioid filling. Interestingly, there was a high rate of opioid refills (43.9%) in this patient population. However, these numbers are similar to the rate reported by Sabatino et al. in their study of prescribing patterns after THA. This analysis is also limited to 2010–2018 since these are the dates available for analysis in the most updated version of PearlDiver. It would be ideal to evaluate trends prior to 2010 as prescription filling has likely changed over a longer period of time than 2010–2018. Nonetheless, the earliest state-wide opioid-limiting legislation was enacted in 2016, which is well captured in this study. Lastly, we are unable to neatly separate the inter-related influence of national paradigm shifts in opioid prescription patterns from state-specific legislation. However, our analyses suggest that states with legislation have decreased filling volumes compared to states without legislation. Specifically, states mandating duration and volume limits had the largest decreases in opioid prescribing.

## Conclusions

In conclusion, opioid filling after primary THA has decreased considerably in the United States since 2010. This descriptive study suggests part of this decrease could be related to opioid-specific state legislation. Although definitive conclusions cannot be drawn from these analyses, our finding suggest that states without opioid legislation may benefit from opioid legislation to decrease opioid overprescribing. These descriptive data also could be used to generate hypotheses for future studies that use more rigorous designs to determine the effect of policy and opioid prescription.

### Supplementary Information

**Additional file 1: Table S1.** CPT, ICD-9, and ICD-10 code ranges used to identify patient cohorts.

**Additional file 2: Table S2.** Opioid legislation types and enactment dates for each state. The mean date of legislation (9/10/2017) was used for states without opioid legislation.

## Figures and Tables

**Fig. 3:**
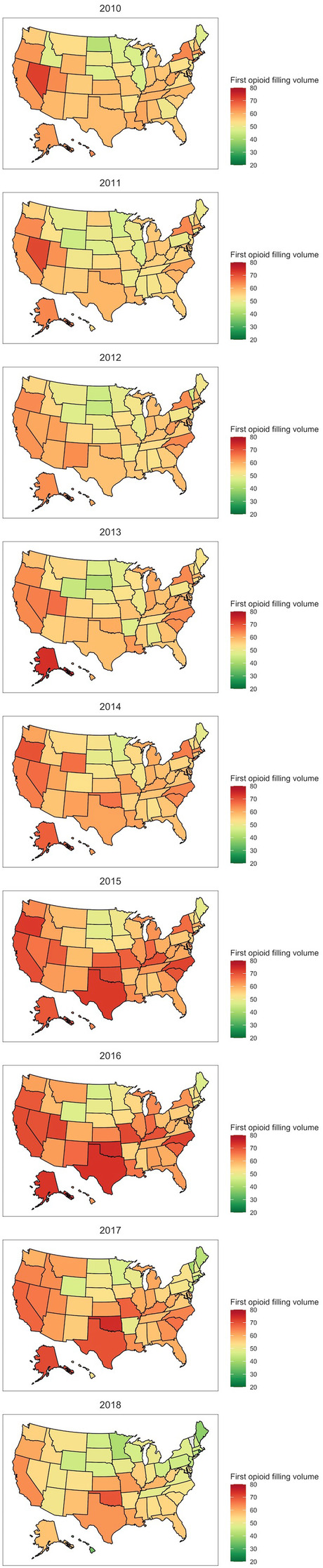
State-level initial prescription oxycodone 5-mg equivalent filling volume from 2010 to 2018

**Fig. 4:**
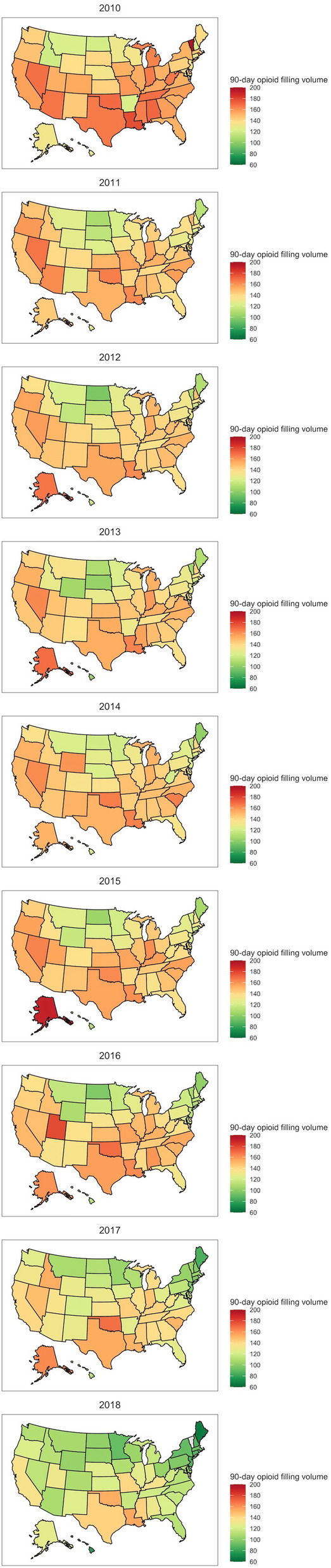
State-level 90-day cumulative prescription oxycodone 5-mg equivalent filling volume from 2010 to 2018

## Data Availability

The data that support the findings of this study are available from PearlDiver, Inc. but restrictions apply to the availability of these data, which were used under license for the current study, and so are not publicly available. Data are however available from the authors upon reasonable request and with permission of PearlDiver, Inc.

## Abbreviations

THA: total hip arthroplasty
AAHKS: American Association of Hip and Knee Surgeons
STROBE: Strengthening the Reporting of Observational Studies in Epidemiology
CCI: Charlson comorbidity index
CDC: Centers for Disease Control
MME: morphine milligram equivalents
30PRE-90POST: 1-month pre-operative to 3-months post-operative time period
ANOVA: analysis of variance
OE: oxycodone 5-mg equivalent
CPT: Current Procedural Terminology
ICD: International Classification of Diseases
