# Supplementary material for: Opioid legislation and narcotic filling in total hip arthroplasty: descriptive study of time and state-level trends in the United States
Source: Subst Abuse Treat Prev Policy. 2021 Sep 28;16:75. doi: 10.1186/s13011-021-00410-w (PMC8477542; doi:10.1186/s13011-021-00410-w)
Supplement: Supplementary file 1 — Additional file 1: Table S1. CPT, ICD-9, and ICD-10 code ranges used to identify patient cohorts. [file 13011_2021_410_MOESM1_ESM.docx]

Additional file 1: Table S1. CPT, ICD-9, and ICD-10 code ranges used to identify patient cohorts.

| **Factor** | **CPT codes** | **ICD-9 code range** | **ICD-10 code range** |
| --- | --- | --- | --- |
| THA | 27130 | 81.51 | 0SR9019, 0SR901A, 0SR901Z, 0SR9029, 0SR902A, 0SR902Z, 0SR9039, 0SR903A, 0SR903Z, 0SR9049, 0SR904A, 0SR904Z, 0SR9069, 0SR906A, 0SR906Z, 0SR90J9, 0SR90JA, 0SR90JZ, 0SRB019, 0SRB01A, 0SRB01Z, 0SRB029, 0SRB02A, 0SRB02Z, 0SRB039, 0SRB03A, 0SRB03Z, 0SRB049, 0SRB04A, 0SRB04Z, 0SRB069, 0SRB06A, 0SRB06Z, 0SRB0J9, 0SRB0JA, 0SRB0JZ |
| Obesity | n/a | 278.00 - 278.03, V85.20 -V85.49, V45.86 | E66.0 - E66.9 |
| Hip fracture | n/a | 820.0 - 820.9 | S72.0 - S72.26 |
