# Supplementary material for: Opioid legislation and narcotic filling in total hip arthroplasty: descriptive study of time and state-level trends in the United States
Source: Subst Abuse Treat Prev Policy. 2021 Sep 28;16:75. doi: 10.1186/s13011-021-00410-w (PMC8477542; doi:10.1186/s13011-021-00410-w)
Supplement: Supplementary file 2 — Additional file 2: Table S2. Opioid legislation types and enactment dates for each state. The mean date of legislation (9/10/2017) was used for states without opioid legislation. [file 13011_2021_410_MOESM2_ESM.docx]

Additional file 2: Table S2. Opioid legislation types and enactment dates for each state. The mean date of legislation (9/10/2017) was used for states without opioid legislation.

| **State** | **Legislation type** | | **Enactment date** | |
| --- | --- | --- | --- | --- |
| *Duration* |  | |  | |
| KY | 3-day duration | | 6/29/17 | |
| MN | 4-day duration | | 5/30/17 | |
| AZ | 5-day duration | | 1/26/18 | |
| NJ | 5-day duration | | 2/15/17 | |
| AK | 7-day duration | | 7/25/17 | |
| CT | 7-day duration | | 5/27/16 | |
| DE | 7-day duration | | 4/1/17 | |
| HI | 7-day duration | | 7/3/17 | |
| IN | 7-day duration | | 7/1/17 | |
| LA | 7-day duration | | 8/1/17 | |
| MA | 7-day duration | | 3/14/16 | |
| MI | 7-day duration | | 12/1/17 | |
| NC | 7-day duration | | 1/1/18 | |
| NH | 7-day duration | | 1/1/17 | |
| NY | 7-day duration | | 6/22/16 | |
| PA | 7-day duration | | 11/2/16 | |
| UT | 7-day duration | | 3/22/17 | |
| VA | 7-day duration | | 3/15/17 | |
| WV | 7-day duration | | 3/27/18 | |
| *Volume* |  | |  | |
| VT | 24-32 MME per day | | 7/1/17 | |
| WA | 42 tablets | | 7/23/17 | |
|  | |  | |  |
| OH | 30 MME per day, 7-day duration | | 8/31/17 | |
| RI | 30 MME per day, 7-day duration | | 6/28/16 | |
| NV | 90 MME per day, 14-day duration | | 6/16/17 | |
| ME | 100 MME per day, 7-day duration | | 4/19/16 | |
|  | |  | |  |
| MD | No specific duration or amount | | 5/25/17 | |
| OR | No specific duration or amount | | 10/25/18 | |
|  | |  | |  |
| AL | none | | 9/10/17 | |
| AR | none | | 9/10/17 | |
| CA | none | | 9/10/17 | |
| DC | none | | 9/10/17 | |
| GA | none | | 9/10/17 | |
| ID | none | | 9/10/17 | |
| IL | none | | 9/10/17 | |
| KS | none | | 9/10/17 | |
| MO | none | | 9/10/17 | |
| MS | none | | 9/10/17 | |
| MT | none | | 9/10/17 | |
| ND | none | | 9/10/17 | |
| NM | none | | 9/10/17 | |
| SD | none | | 9/10/17 | |
| WI | none | | 9/10/17 | |
| WY | none | | 9/10/17 | |
